# Large-scale phenotyping of 1,000 fungal strains for the degradation of non-natural, industrial compounds

**DOI:** 10.1038/s42003-021-02401-w

**Published:** 2021-07-15

**Authors:** David Navarro, Delphine Chaduli, Sabine Taussac, Laurence Lesage-Meessen, Sacha Grisel, Mireille Haon, Philippe Callac, Régis Courtecuisse, Cony Decock, Joëlle Dupont, Florence Richard-Forget, Jacques Fournier, Jacques Guinberteau, Christian Lechat, Pierre-Arthur Moreau, Laetitia Pinson-Gadais, Bernard Rivoire, Lucile Sage, Stéphane Welti, Marie-Noëlle Rosso, Jean-Guy Berrin, Bastien Bissaro, Anne Favel

**Affiliations:** 1 INRAE, Aix Marseille Univ., BBF, UMR1163, Marseille, France; 2 INRAE, Aix Marseille Univ., CIRM-CF, Marseille, France; 3 INRAE, MYCSA, UR1264, Villenave d’Ornon, France; 4 Faculté de Pharmacie Lille, Université de Lille, LGCgE, ER4, Lille, France; 5 Mycothèque de l’Université Catholique de Louvain (MUCL), Earth and Life Institute, Microbiology, Louvain-la-Neuve, Belgium; 6 Institut de Systématique, Evolution et Biodiversité, ISYEB – UMR 7205 – CNRS, MNHN, UPMC, EPHE, Muséum National d’Histoire Naturelle, Sorbonne Universités, Paris, France; 7 Las Muros, Rimont, France; 8 Ascofrance, Villiers-en-Bois, France; 9 Société Linnéenne, Lyon, France; 10 Université Grenoble Alpes, LECA, UMR UGA-USMB-CNRS 5553, CS 40700, Grenoble, France

**Keywords:** Environmental biotechnology, Fungal biology

## Abstract

Fungal biotechnology is set to play a keystone role in the emerging bioeconomy, notably to address pollution issues arising from human activities. Because they preserve biological diversity, Biological Resource Centres are considered as critical infrastructures to support the development of biotechnological solutions. Here, we report the first large-scale phenotyping of more than 1,000 fungal strains with evaluation of their growth and degradation potential towards five industrial, human-designed and recalcitrant compounds, including two synthetic dyes, two lignocellulose-derived compounds and a synthetic plastic polymer. We draw a functional map over the phylogenetic diversity of *Basidiomycota* and *Ascomycota*, to guide the selection of fungal taxa to be tested for dedicated biotechnological applications. We evidence a functional diversity at all taxonomic ranks, including between strains of a same species. Beyond demonstrating the tremendous potential of filamentous fungi, our results pave the avenue for further functional exploration to solve the ever-growing issue of ecosystems pollution.

## Abstract (web summary)

Navarro et al. present a culture-based approach to the degradation of industrial products and by-products by assessing >1,000 fungal strains. Using growth-assay-screening and a large sample of fungal phenotypes, they explore the functional differences across strains and species to demonstrate the potential of filamentous fungi in breaking down dyes and industrial by-products with lignocellulose and plastic bases.

## Introduction

Over the past 200 years, humankind has engineered processes and commodities that have greatly contributed to the life standards we enjoy today. However, this progress has come with a cost we are just beginning to assess. Plastics, dyes, and additives are widely used in the food, agricultural, medical, transport, textile, cosmetic, and electronic industries. These compounds have been designed to be resistant to degradation and are thus difficult to recycle, which poses today major challenges. The emerging bioeconomy^1^ and in particular white biotechnologies are set to play a cornerstone role in future societies, notably through the discovery and engineering of efficient biocatalysts (enzymes and microorganisms)^2^. The fungal kingdom contains an estimated diversity ranging between 2.2 and 3.8 million species^3^ and is considered as a remarkable reservoir of enzymes and biotechnological solutions^4^. The diversity of fungal lifestyles (saprobic, symbiotic, or parasitic), together with the diverse properties of their hosts (e.g., plants, insects, animals), are key drivers of the evolution of their extraordinary biocatalytic potential. Notably, plant-decaying filamentous fungi display the unique capacity to degrade efficiently lignocellulose, a notoriously recalcitrant biopolymer. The underlying biochemical strategies have inspired many industrial processes for plant biomass valorization into fermentable sugars and other biomolecules for second-generation biofuels, bioproducts, and biomaterials^5–7^. Noting the analogy of challenges intrinsic to both lignocellulosic and human-designed polymers (e.g., crystallinity, insolubility, heterogeneity, toxicity), fungi, and enzymes thereof represent a yet untapped reservoir of biological tools to be discovered and harnessed to face new challenges (e.g., plastics bioconversion).

As defined in 2001 by the Organization for Economic Cooperation and Development, the creation and maintenance of Biological Resource Centres (BRC) is of great importance to the scientific community to tackle global challenges of modern societies^8^. In France, the CIRM-CF (Centre International de Ressources Microbiennes – Champignons Filamenteux, https://www.cirm-fungi.fr) is a French BRC created in 2006 and gathering representatives of the fungal diversity and associated molecular and physiological information. The CIRM-CF preserves and studies not only >2800 fungal original strains of biotechnological interest, mainly saprobic species growing on plant materials, but also strains from polluted agro-industrial sites.

Here we present the first large-scale phenotyping study to assess the potential of this fungal collection toward five non-natural compounds used at industrial scales, including dyes from the textile industry, lignosulfonates (LGSs) from the pulp and paper industry, soluble polyurethane from the plastic industry, and microcrystalline cellulose used in the food, pharmaceutical, and cosmetic industries. We describe the functional phenotyping (growth and degradation assays) of >1000 strains belonging to 26 orders, 78 families, and 231 different genera, according to the Mycobank Database^9–11^. The functional diversity was analyzed in regard to the ecology of the species and the taxonomy at the family, species, or intraspecies levels. Our work unveils the high biotechnological potential of fungal diversity for the development of sustainable solutions to preserve our planet.

## Results

### Building of the CIRM-CF fungal collection

**Fig. 1:**
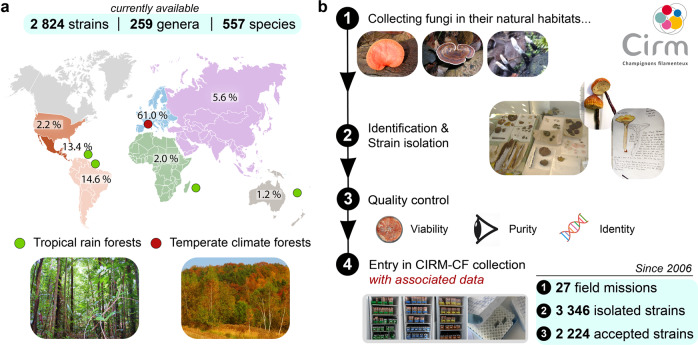
Diversity and enrichment strategy of the CIRM-CF fungal collection. **a** Overview of the taxonomic and geographic diversity of the 2824 (as of May, 2021) available fungal strains preserved in the CIRM-CF. **b** Workflow of strains enrichment in the CIRM-CF collection from 2006 to 2020, with fungi coming from mainly France and its overseas territories. The first step consists in collecting fungi in their natural habitat. Second, each collected sample is registered and identified by expert mycologists. The strain isolations are directly performed in a field laboratory, by the CIRM-CF or by associated mycologists from Universities and French learned societies. Finally, the viability, the purity, and the identity of each strain are checked. When the strains are viable, pure, and when the molecular information is in accordance with the morphological identification, the strains can enter the CIRM-CF collection.

As of May 2021, the CIRM-CF collection contains 2824 fungal strains, from 259 genera and 557 species (Fig. 1a). The collection results from our efforts deployed since 2006 to acquire and preserve the fungal diversity living in French territories (Fig. 1b), through field collection (in natural habitats) and strain deposits by mycologists. As shown in Fig. 1b, the CIRM-CF took part in 27 field collecting expeditions in tropical rainforests (mainly French Guiana, Martinique, Guadeloupe) as well as in temperate forests. As a result, in collaboration with a consortium of expert mycologists (from Universities and learned societies), we have carried out the macro- and micro-morphological identification and strain isolation of 3346 candidate strains. In order to ensure that high-quality BRC standards are met, we checked the purity and the viability of candidate strains by three successive sub-cultures before entering them in the collection. Moreover, we systematically checked strain identity (initially deduced from morphological identification keys) using molecular authentication such as the genotyping of one barcoding gene (usually ITS1-5.8S-ITS2). After applying these selection filters, we rejected about one-third of isolated strains due to (i) impurity, (ii) poor viability, or (iii) when the molecular information was not in agreement with specimen morphological identification. This elevated exclusion rate highlights the technical hurdles and challenges pertaining to proper cultivation and/or isolation of pure fungal strains from natural habitats. After authentication, the fungal strains are maintained with 3 different storage modes (cryopreservation under liquid nitrogen, water, and oil-submerged cultures at 4 °C) in different locations and the viability of the cryopreserved cultures are checked after 6 months.

From the complete collection of the CIRM-CF, a total of 1031 fungal strains were selected for this functional study. These 1031 strains were selected to proportionally represent the taxonomic diversity of the strains available in the CIRM-CF collection. Precisely, this set encompasses 400 species, 99% from the *Ascomycota* and *Basidiomycota* phyla, and spanning 26 orders, 78 families (43 *Basidiomycota*, 31 *Ascomycota*, and 4 *Mucoromycota* families), and 231 genera (135 *Basidiomycota*, 91 *Ascomycota*, 5 *Mucoromycota* genera; Supplementary Data 1 and Supplementary Table 1). In all, 68% of these strains were collected in French territories, 40% of which coming from overseas territories (mainly French Guiana and Martinique). Of note, fungal orders for which at least 10 strains were tested (shown in bold characters in Supplementary Table 1) encompass 365 different species. Furthermore, although the strain sampling of some orders could appear as downsized (e.g., 20 vs 392 strains in the *Gloeophyllales* and *Polyporales* orders, respectively), the diversity sampling is still ensured since these seemingly downsized orders usually contain less families (e.g., 1 vs 18 families, respectively)^12,13^. We underscore that the CIRM-CF collection is dedicated to filamentous fungi able to decay plant biomass transformation, the represented fungi are thus almost exclusively saprobic (growing on wood particularly).

### Set-up of the large-scale multi-phenotyping

**Fig. 2:**
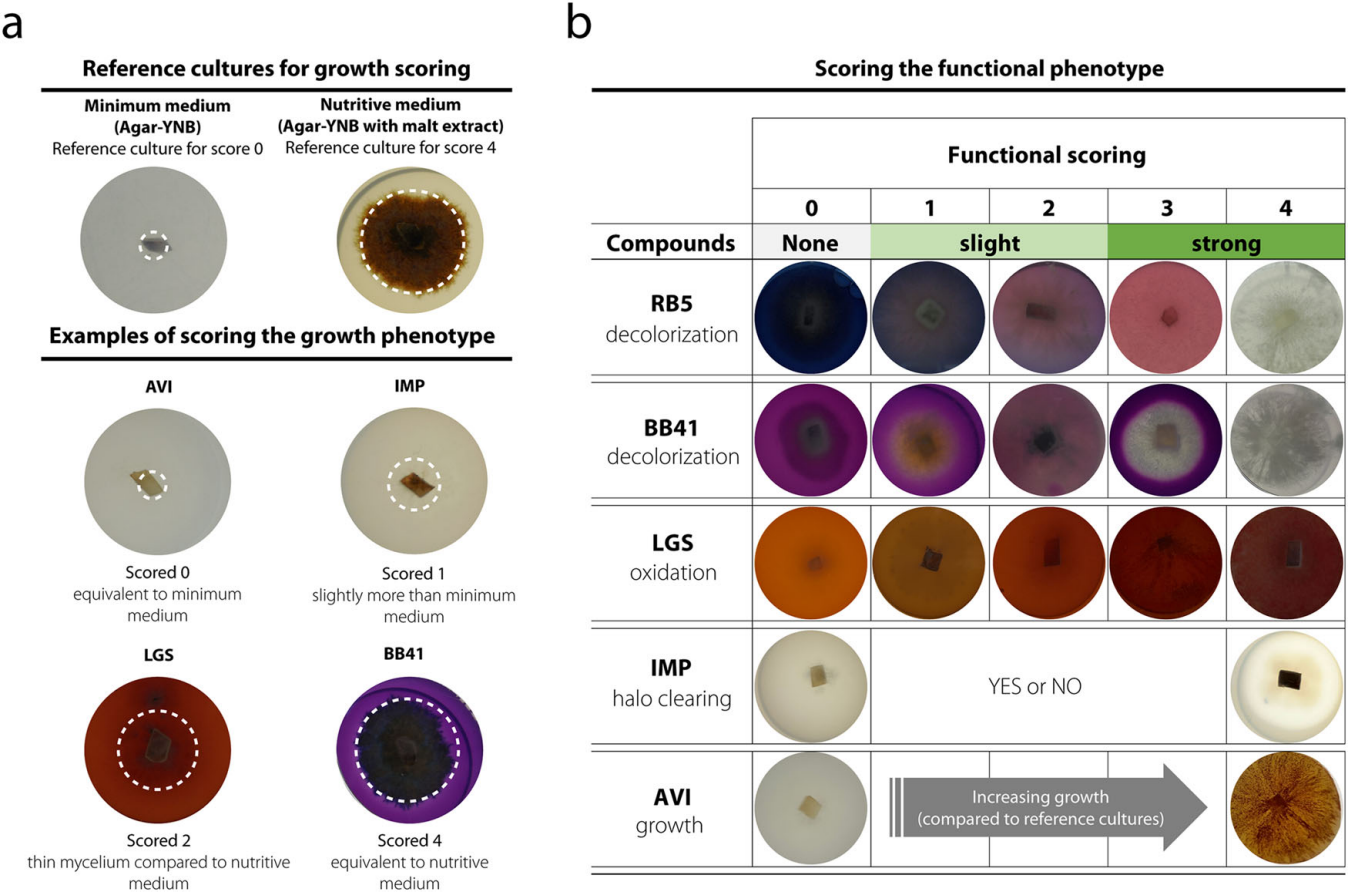
Functional screening methodology. **a** The growth phenotype was scored by comparing the mycelium growth in a given condition (i.e., agar-YNB + tested compound) vs the two following controls setting the boundaries of the growth scale: agar-YNB plate (score 0) and malt agar plate (score 4). Note that for RB5 and BB41 the agar plates contained 0.5% malt (vs 1.5% malt in positive control plate) to sustain growth. The white dotted circle indicates the fungal growth diameter (which is not always visible on pictures). **b** Functional phenotype scores (ranging from 0 to 4) reflect the extent of decolorization (on the industrial dyes Reactive Black 5 (RB5) and Basic Blue 41 (BB41)), browning upon phenol oxidation (on lignosulfonate (LGS)), clearing halo formation (on the soluble polyurethane Impranil® DLN (IMP)) or growth (on Avicel (AVI)). A score of 0 means no decolorization/degradation/oxidation. The pictures show examples for the different scores.

To assess the degrading potential of the fungal strains toward molecules engineered by mankind (Fig. 2), we selected five compounds known to be highly recalcitrant to degradation^14–17^: two different synthetic azo dyes, Reactive Black 5 (RB5) and Basic Blue 41 (BB41), which are among the most commonly used dyes in the textile industry, and thus major environmental toxic pollutants; soluble LGS as a pollutant coproduct from the pulp and paper industry; Impranil® DLN-SD (IMP) as a soluble polyurethane used in plastic and textile industries; and microcrystalline cellulose (Avicel®PH-101 (AVI)), a recalcitrant refined wood pulp, notably widely used in the food industry as texturizer (Supplementary Fig. 1). For each strain, a six-well plate was used to assess simultaneously the different growth conditions (Fig. 2a), including (i) a positive control culture on malt medium and (ii) each of the five tested compounds suspended in agar-yeast nitrogen base (YNB) minimum medium (except for RB5 and BB41, vide infra). Of note, a “negative” control culture on agar-YNB minimum medium (devoid of additional carbon source) was systematically assessed in parallel. After an incubation period of 13 days at 25 °C, different phenotypes were observed: the decolorization of RB5 and BB41, phenol oxidation of LGS, clearing halo formation of IMP, and growth on AVI. The biosorption (i.e., absorption on the mycelium) of the dyes was not considered as decolorization. To obtain semi-quantitative information on the efficiency of degradation, we attributed fungal growth scores (from 0 to 4; Fig. 2a) and degradation scores (from 0 to 4; Fig. 2b), henceforth called “functional phenotype scores,” as follows: for fungal growth (mycelium diameter and density) scoring, a score of 0 was attributed when no growth difference was observed between a given tested condition and the agar plate control (Fig. 2a). When growth was similar to cultures on malt agar plate (positive control), a score of 4 was attributed. Intermediate growth was attributed intermediate score (from 1 to 3). Regarding functional phenotype scores (Fig. 2b), scores for azo dye (RB5 and BB41) decolorization and LGS phenol oxidation were 0 (no decolorization/oxidation), 1 (light), 2 (medium), 3 (strong), or 4 (maximum), whereas scores for clearing halo formation on IMP were 0 (negative) or 4 (positive). For Avicel, the functional phenotype score was the growth score (raw data are available in Supplementary Data 2).

**Fig. 3:**
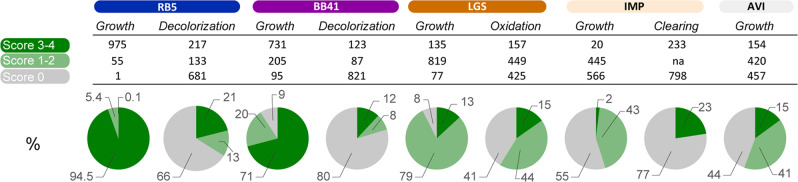
Global phenotype scoring of the 1031 filamentous strains. The figure shows for each substrate the number of strains displaying growth and either decolorization (for RB5 and BB41), or oxidation (for LGS) or clearing (for IMP), clustered by score intensity (0, 1–2, and 3–4), see Fig. 2 legend for details on score definition. The pie charts show the relative distribution of scores categories (100% = 1031 strains). AVI Avicel, BB5 Basic Blue 41, IMP Impranil, LGS lignosulfonate, na not applicable, RB5 Reactive Black 5.

For RB5 and BB41, preliminary tests indicated that the minimum medium needed to be supplemented with malt extract, as otherwise no fungal growth could be observed. Therefore, for these two substrates fungal growth cannot be associated with RB5 and BB41 being used as carbon source. Most fungal strains were able to grow (scores 1 to 4) on malt agar plates containing RB5 (99.9%) and BB41 (91%) (Fig. 3). Yet, the relatively inferior growth (scores ≤2) for a significant number of strains (5.5% for RB5 and 29% for BB41), compared to the positive control and scored 4 strains, points at inhibitory growth properties of these azo-dyes, particularly for BB41. Furthermore, growth was clearly not correlated to decolorization potential as only 34% (RB5) and 20% (BB41) of the strains significantly achieved decolorization (Fig. 3). Note that biosorption hits were excluded from the decolorizing strain count. Regarding LGS, most strains (92%) were able to grow, albeit the growth extent remained limited as only 13% reached the scores 3 and 4, most probably due to the difficulty to assimilate LGS that was used as sole carbon source in the culture medium. Yet, 59% of the strains managed to oxidize phenols from LGS (observed by browning). As to the crystalline cellulose, a substrate that could be expected to be readily degraded by saprotrophic fungi, only 15% of strains succeeded to significantly grow. Regarding IMP, our large-scale phenotyping experiment allowed to evidence a few set of strains (2%) showing a significant growth (score ≥3) (and 43% of the strains showed a limited growth), indicating that IMP was the most recalcitrant compound tested in the present study. Nonetheless, 23% of the strains succeeded to form a clearing halo, underlying that limited growth could be sufficient to produce degrading enzymes.

### Functional phenotypes are not correlated with growth capacities

**Fig. 4:**
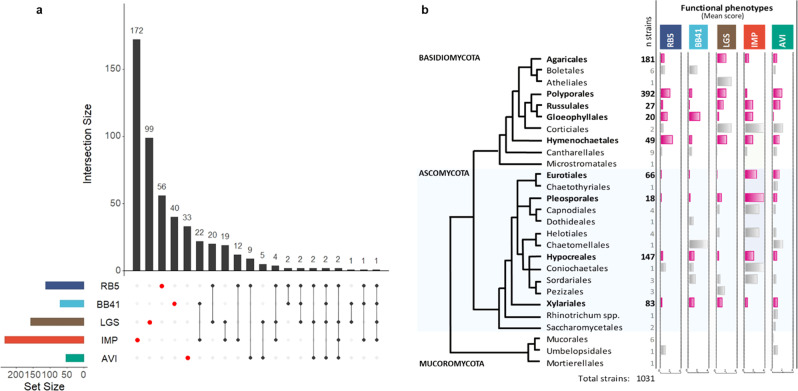
Substrate-wise and phylogeny-wise distribution of the phenotypic scores of the 1031 tested fungal strains. **a** UpSet plots illustrating quantitative intersection of the sets of strains showing the highest score (score 4) for each functional phenotype (the colored bars on the left-hand side indicate the total number of strains that scored a 4 for each compound). For instance, looking at IMP, out of 233 “score 4” strains, 172 scored a 4 exclusively on IMP (and not on any other compound), while 22 scored a 4 on both IMP and BB41. The figure shows that high scoring strains with a single phenotype represent the majority. **b** Mean scores of multi-phenotyping of 1031 natural strains belonging to 26 fungal orders mapped onto the phylogenetic tree. The numbers of analyzed strains (*n* samples) are indicated in the first column. Horizontal histograms show the mean scores for each fungal order for each phenotype, ranging from 0 to 4. Note that these scores reflect a functional phenotype (i.e., decolorization (RB5, BB41), oxidation (LGS), degradation (IMP), or growth (AVI)). Growth phenotypes for all strains/compounds couples are provided in Supplementary Data 2. Histograms colored in pink indicate orders for which more than ten strains were analyzed (otherwise shown in gray). The topology of the tree was built according to recent phylogenies of Fungi^57–59^.

As mentioned above, we did not observe any correlation between growth capacities and (i) decolorization of azo dyes, (ii) oxidation of LGS, or (iii) clearing of IMP (Supplementary Fig. 2a). This result suggests that extensive fungal biomass development is not required for degrading the targeted compounds. In an attempt to identify potential correlations between the phenotypes, we focused our attention on the best-performing strains (with the highest score of 4) for each phenotype (Supplementary Data 2) and generated an UpSet plot (Fig. 4a). Remarkably, most of the best-performing strains were shown to have a single, preferred target compound, with little overlap observed between functional phenotypes (Fig. 4a). Strikingly, this observation was also true for RB5 and BB41, two azo dyes characterized by different chemical structures and number of azo bonds (Supplementary Fig. 1). This lack of correlation between phenotypes suggests that the best-performing strains make use of specialized enzymatic activities not universally shared. Regarding the phylogenetic distribution of the observed phenotypes (Fig. 4b), for orders in which >10 strains could be characterized (shown in bold characters/pink histograms in Fig. 4b), the following general trend stood out: *Basidiomycota* appear to degrade or modify a broader range of compounds than *Ascomycota*. RB5 was predominantly decolorized by *Basidiomycota*, whereas BB41 was decolorized by both *Basidiomycota* and *Ascomycota* (mainly *Gloeophyllales*, *Hypocreales*, and *Xylariales* orders). The oxidation of LGS was mainly observed for *Basidiomycota*, although some *Ascomycota* showed relatively high mean scores (*Pleosporales*, *Hypocreales*, *Xylariales*). The clearing activity on IMP was observed in a large array of fungal orders with *Ascomycota* fungi (*Pleosporales*, *Eurotiales*, *Hypocreales*) displaying the highest scores. Finally, the *Polyporales* and *Russulales* orders, belonging to *Basidiomycota* phylum, showed the best mean scores for growth on microcrystalline cellulose.

### Fungi display a great functional diversity at every taxonomic rank

**Fig. 5:**
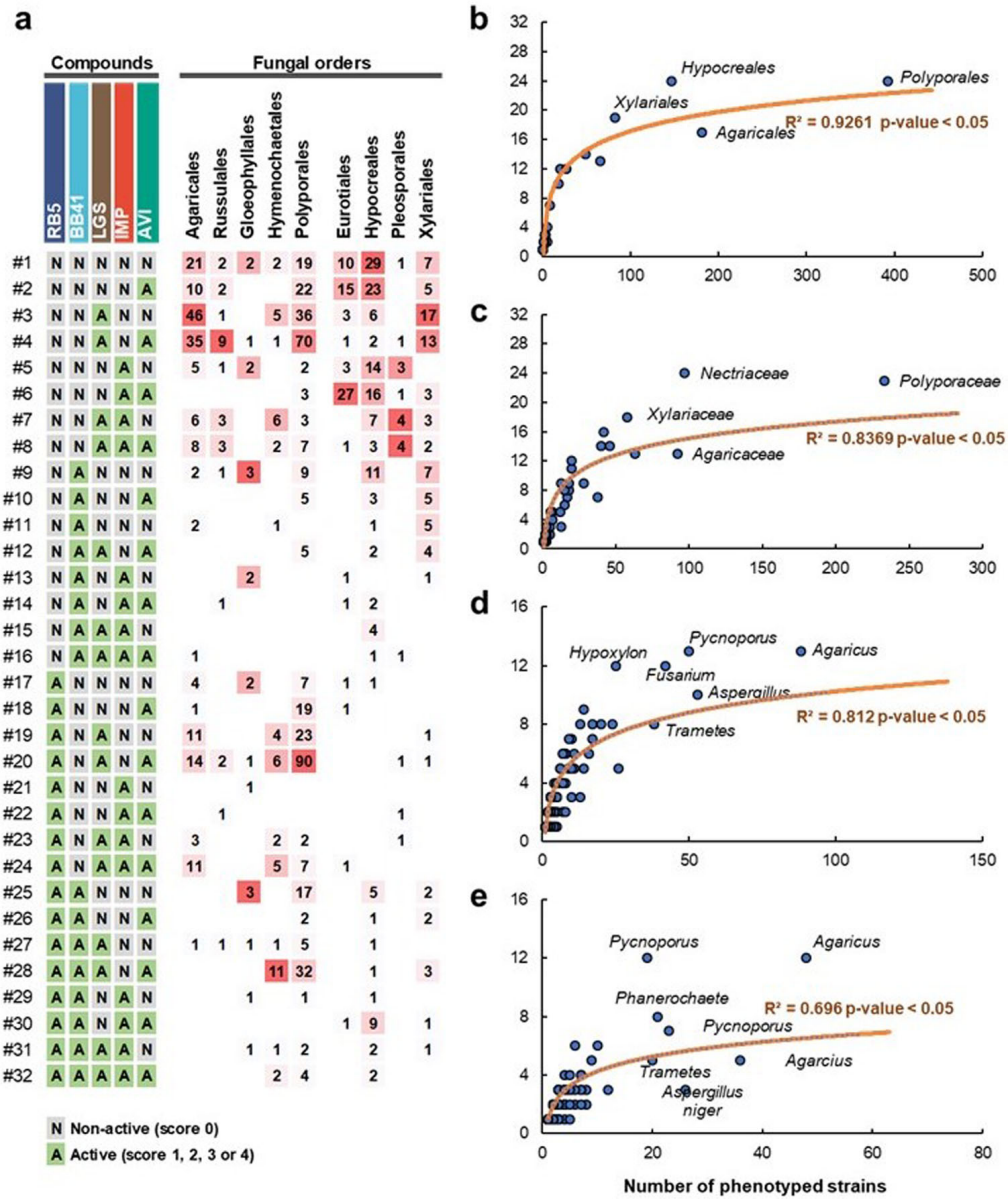
Overview of the functional diversity at different taxonomic levels. **a** Distribution of functional profiles at the order level. The two-level categorization of non-active (N) and active (A) substrate–strain couples evaluated on 5 different substrates yields a theoretical maximum of 32 profiles. For each fungal order, the numbers provided in the matrix represent the number of strains with a given profile (for each fungal order, a color gradient from white (only one strain) to red (maximum number of strains) has been applied to show the distribution of functional profiles in the order). As an example, 46 strains from the *Agaricales* order displayed the profile #3 (LGS oxidation only). **b**–**e** Logarithmic regression between the number of functional profiles observed (*x* axis) within a taxonomic rank (indicated in the figure) and the number of analyzed strains (*y* axis) in this rank, at the order (**b**), family (**c**), genus (**d**), and species (**e**) levels.

The global analysis presented herein above highlighted that most of the best-performing strains display a main phenotype, although minor “activity” toward other targets could be detected. To probe the order/species-dependent functional diversity, we categorized each target compound/strain couple as either non-active (“N,” i.e., when score was 0) or as active (“A,” i.e., for scores from 1 to 4). Given that 5 compounds were tested, this 2-level categorization yields in theory 2^5^ possible phenotype combinations (Fig. 5a). Interestingly, the 32 theoretically possible functional profiles were all observed at least once, albeit profiles with single and dual phenotypes were preponderant. We observed that some profiles were more frequent than others such as the functional profile #3 (LGS oxidation) and #4 (LGS oxidation and growth on microcrystalline cellulose). Taking the *Russulales* order as an example, while profile #4 gathers one-third of the tested strains, the remaining two-thirds are distributed across 11 different profiles, indicating the presence of a significant functional diversity within a single order.

To delineate the “strain effect” on the observed functional diversity, we then analyzed to which extent the number of tested strains impacted the number of detected functional profiles (Fig. 5b–e). Unsurprisingly, at the order level, the higher the number of tested strains, the more the functional profiles were observed (Fig. 5b). We also observed a similar correlation at the family (Fig. 5c) and genus (Fig. 5d) levels. Astonishingly, a high functional diversity was also noticed between strains belonging to a same species (Fig. 5e). Depending on the species, 3–12 distinct profiles were observed when >10 strains of the same species were screened. For instance, 12 distinct profiles were observed with the phenotyping of 19 strains of *Pycnoporus cinnabarinus* or the phenotyping of 49 strains of *Agaricus bisporus*. Using the non-linear regressions shown in Fig. 5b–e, and provided that good enough taxonomic coverage/species diversity is ensured, we estimated the minimal set of strains to be screened to cover a functional diversity as large as possible to be 150, 75, 40, and 20 strains at the order, family, genus, and species level, respectively.

### A roadmap for the selection of fungal families for dedicated applications

**Fig. 6:**
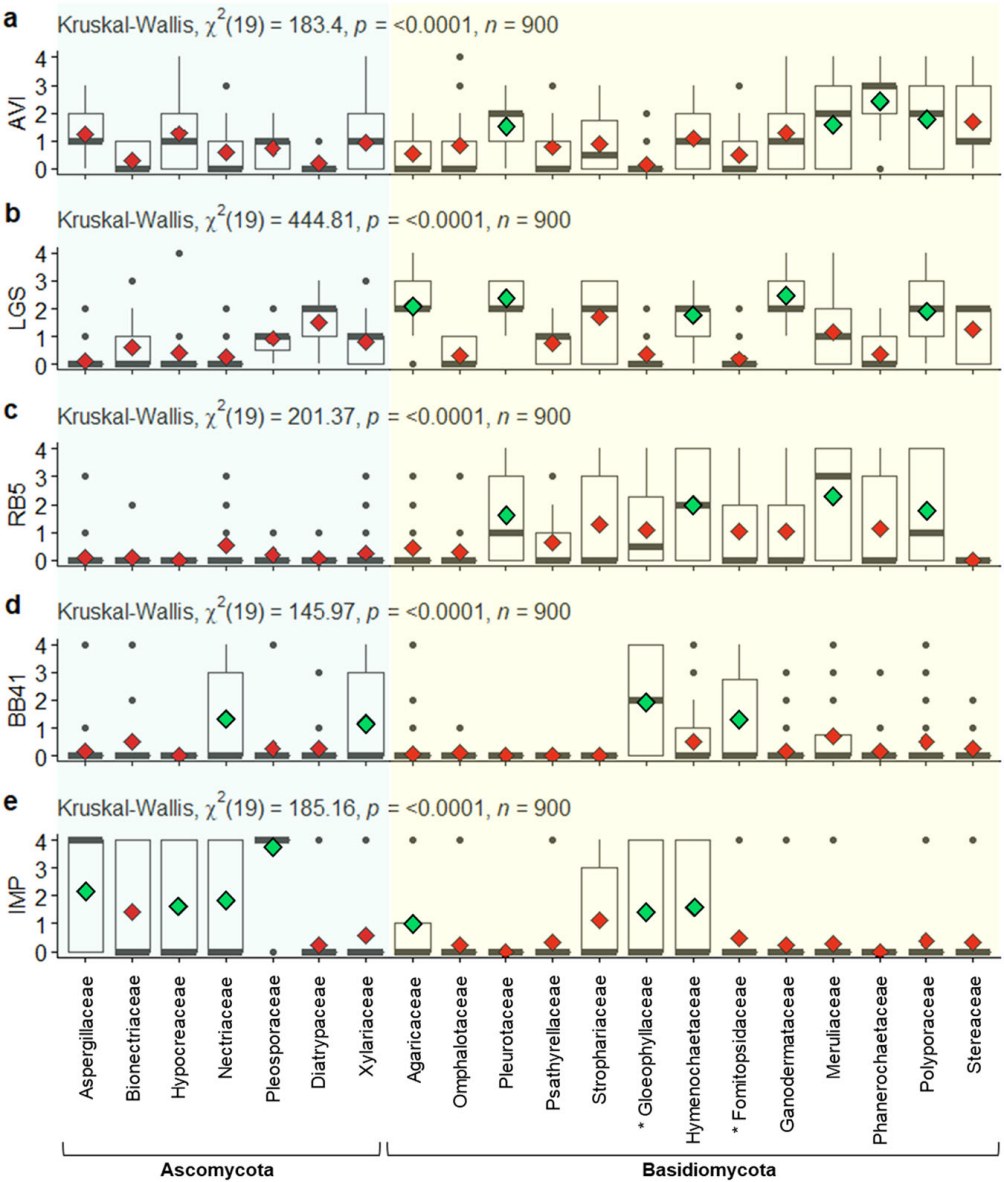
Box plots showing the fungal family-dependent distribution of phenotypic scores. For each fungal family, and each target compound (**a**–**e**), the median score (thick gray bars) and mean score (red or green diamonds) are shown. For a given target compound, green diamonds show families with a mean score significantly higher than other families. Outliers are represented by dots. Families of brown-rot fungi are marked by an asterisk. For each target compound, non-parametric Kruskal–Wallis test indicates (*p* value and *χ*^2^) significative differences between fungal families. Wilcoxon signed-rank test were computed to compare paired data (i.e., family to family; *p* values are shown in Supplementary Data 3).

Among the wide diversity offered by the fungal kingdom, identifying the most suitable fungal family(ies) for a dedicated application is a true challenge. Here we set off to provide knowledge-based selection guidelines regarding the degradation of the five selected industrial compounds and applications thereof. To this end, we analyzed the statistical distribution of the functional phenotype scores within fungal families with satisfying diversity coverage (i.e., >10 tested strains), representing a total of 900 fungal strains (Fig. 6 and Supplementary Data 3). Horizontal reading of Fig. 6 shows that some compounds are preferentially targeted by specific fungal families (e.g., RB5 by *Basidiomycota* families) while vertical analysis informs that some fungal families display a marked preference for specific industrial compounds (e.g., *Phanerochaetaceae* with AVI). Strikingly, almost all families revealed the presence of high-score outliers, highlighting that even fungal families with seemingly overall poor activity on a particular compound can contain a few efficient strains.

In detail, regarding microcrystalline cellulose, out of the 154 strains that showed growth, 142 belong to the *Basidiomycota* phylum (Fig. 6a), with a significantly higher prevalence of strains from the *Phanerochaetaceae*, *Polyporaceae*, and *Meruliaceae* families. We underscore that strains from the phlebioid clade, within the order Polyporales (*Phanerochaetaceae*, *Irpicaceae*, *Meruliaceae*)^12,18^, show the highest mean scores of growth on microcrystalline cellulose. For *Ascomycota*, the *Hypocreaceae*, *Aspergillaceae*, and *Xylariaceae* families stood out with numerous strains able to slightly grow on microcrystalline cellulose (median score ≤2).

Concerning LGS oxidation, families from lignicolous *Basidiomycota* showed relatively good mean scores, with highest prevalence being observed for strains from the *Ganodermataceae*, *Pleurotaceae*, *Agaricaceae*, *Polyporaceae*, and *Hymenochaetaceae* families (Fig. 6b). In contrast, the mean scores obtained by brown-rot families (*Gloeophyllaceae*, *Fomitopsidaceae*) were very low. This observation is consistent with the acknowledged limited abilities of brown-rot fungi to degrade or modify lignin in nature (see “Discussion”).

Regarding the decolorization of RB5 (Fig. 6c), stark differences between the fungal phyla were observed, *Basidiomycota* being in general much more efficient than *Ascomycota*. In particular, the *Basidiomycota* families *Pleurotaceae* (69% of tested *Pleurotaceae*), *Meruliaceae* (66%), *Hymenochaetaceae* (63%), and *Polyporaceae* (56%) showed the strongest prevalence for decolorizing RB5. The fact that families of wood decaying *Basidiomycota* were overall the most efficient for RB5 decolorizing is in line with previous observations made on synthetic dyes^19^. For *Ascomycota*, a few strains were outstanding, in particular among the *Fusarium* species (*Nectriaceae*). Strikingly, the taxonomic distribution of phenotypes observed on the second tested dye, BB41, was totally different from that of RB5 (Fig. 6d), *Gloeophyllaceae*, *Nectriaceae*, *Xylariaceae*, and *Fomitopsidaceae* families displaying the best mean scores.

Finally, concerning the commercial polyester polyurethane IMP, we were surprised to observe that all families except two had at least one strain able to efficiently clear IMP (Fig. 6e). Indeed, Fig. 6e shows outliers in all families, except the *Pleurotaceae* and *Phanerochaetaceae* families. In general, *Ascomycota* were more efficient (higher mean and median scores) than *Basidiomycota* at degrading such artificial polymer. In particular, the *Pleosporaceae* family showed the best mean score, with 14 out of the 15 tested strains clearing IMP with success, followed by the *Aspergillaceae* family that displayed >50% of positive strains. It is noteworthy that genera containing pathogenic species (*Botrytis*, *Sclerotinia*, *Alternaria*, *Phoma*, *Colletotrichum*, *Mycosphaerella*, *Verticillium*, *Trichothecium*, *Fusarium*…) showed a propensity to be good IMP degraders. Yet, some *Basidiomycota* families (in particular *Hymenochaetaceae*, *Gloeophyllaceae*, *Strophariaceae*, *Agaricaceae*) also emerged (because of few strains from *Phellinus* (*sensu stricto*), *Tropicoporus*, *Gloeophyllum*, *Heterobasidion*, *Amylosporus*, *Trichaptum*, *Laetisaria*, *Agrocybe*, and *Agaricus* genera) as potential good candidates to uncover novel polyesterases (see “Discussion”).

## Discussion

Fungi are set to play a cornerstone role in the success of the emerging bioeconomy, in particular to propose innovative biotechnological solutions to pollution issues originating from human-designed compounds. Intensive fungal genome sequencing programs carried out in the past decade teach us that a tremendous diversity in terms of fungal enzyme activities and mechanisms is to be expected^20^. However, functional exploration of such diversity is still very limited and selecting the most appropriate fungal strains for a dedicated application remains a true challenge. Here, by carrying out a large-scale phenotyping of 1031 fungal strains, on 5 different industrial, non-natural compounds, we (i) map the biotechnological potential of filamentous fungi, (ii) demonstrate that functional diversity can be observed down to the intra-species level, and (iii) provide guidelines for the selection of fungal families/species for selected applications. Such endeavor would not have been possible without the existence of fungal BRCs, which, due to a time-consuming and painstaking work, collect, acquire, authenticate, preserve, and finely characterize their biological materials. Compared to other larger or taxonomically more diverse BRCs, such as the CBS-KNAW (Westerdijk Institute, Netherlands), the IBT Fungal collection (DTU, Denmark), the CABI collection (United Kingdom BRC network), or the BCCM/MUCL (Catholic University of Leuven, Belgium), the “CIRM-CF” has the particularity to be a more focused collection, rich in wood-decaying filamentous fungi. While we believe that this collection is well suited to search for activities on recalcitrant (industrial) compounds, it is also likely that the evaluation of other collections, following the methodology outlined here, could reveal alternative interesting taxonomic ranks, especially if different types of compounds are to be targeted.

Our comparative study lays the foundations for future studies insofar as the observed growth and degrading phenotypes allows highlighting strains with promising, yet-to-be explored enzymatic potential. Among our target substrates, we chose two plant biomass-derived compounds, LGS and AVI, serving as proxy of plant components (lignin and cellulose) that are at the heart of the biorefinery. On the one hand, lignin is still seen as a very recalcitrant polymer for which fungal biotechnology can bring solutions for its efficient conversion. On the other hand, microcrystalline cellulose AVI is already used in various fields (e.g., food, pharmaceutical and cosmetic industries) and the search for new strains efficient at its degradation will prove useful in the future circular bioeconomy context. Here we show that white-rot fungi (e.g., *Ganodermataceae*, *Polyporaceae*, *Hymenochaetaceae*) displayed the best scores for LGS oxidation, which is consistent with their abilities to degrade and mineralize lignin in nature^21^. In contrast, brown-rot fungi showed a poor ability to oxidize LGS. This observation can be rationalized by the fact that, unlike white-rot fungi that can mineralize lignin^22^, brown-rot fungi can only modify it, notably via non-enzymatic reactions (e.g., Fenton chemistry)^23,24^. During evolution, brown-rot fungi have lost their lignin-degrading peroxidase genes^25^ and possess only one or two genes coding for non-ligninolytic, low-redox potential general peroxidases^26,27^. Brown-rot fungi also possess on average three laccases^25,28^, which are, however, usually inefficient on syringyl unit, the main constituent of LGS^29,30^. Furthermore, brown-rot fungi (e.g., the *Gloeophyllaceae* and *Fomitopsidaceae* families) hardly thrive on AVI as carbon source, most likely because of their poor enzymatic arsenal related to crystalline cellulose degradation^31,32^.

Synthetic dyes, such as BB41 and RB5, are known to be a serious source of water pollution, as various industries from developing countries (e.g., textile industry) have been reported to often dump their waste streams in lakes, rivers, or in the sea. Fungi represent good candidates for bioremediation strategies insofar as they are considered as microorganisms highly robust and tolerant to high concentrations of pollutants. Here, we showed that despite their poor efficiency on LGS and AVI, brown-rot fungi proved to be able to decolorize synthetic dyes, notably BB41. It is known that fungal laccases (with redox mediators) and peroxidases can bleach RB5^33–35^, whereas only peroxidases have hitherto been shown to discolor BB41^36,37^. Brown-rot fungi being devoid of ligninolytic peroxidases^25^ probably act on BB41 via other systems, involving either Fenton or other types of heme-peroxidases^38^. For instance, chloroperoxidases, which are produced by brown-rot fungi^39,40^, are able to efficiently oxidize synthetic dyes^41^ and lignin^42,43^. More globally, the observed drastically different taxonomic distribution of BB41 vs RB5 decolorizing strains suggests the involvement of (enzymatic) mechanisms not universally shared by the different fungal families.

The afterlife fate of plastics is a major concern of modern societies. Plastics represent a large class of synthetic polymers, including two main categories, namely, thermoplastics (e.g., polyethylene, polystyrene) and thermoset plastics (e.g., polyurethanes). Here we chose IMP as a proxy of the latter category. IMP is a polyester–polyurethane polymer from Bayer Corporation used for textile, leather, and aircraft fabric coatings^44,45^. For this first large-scale study, we chose IMP because its main chain is composed of heteroatoms, making it more susceptible to be degraded by wild-type fungal strains. IMP is an opaque milky suspension that becomes transparent upon degradation by urethanases and/or polyesterases, such as cutinases^46–48^. In our study, *Ascomycota*, and in particular phytopathogenic species, appeared to be more amenable to degrade IMP, which may be related to the capacity of phytopathogenic fungi to penetrate living-plant surfaces via the recruitment of cutinases^49,50^. This observation is consistent with previous studies showing several *Ascomycota* strains able to form a clearing halo from the IMP^46,47,51^. However, the fact that non-pathogenic *Basidiomycota* strains, notably from *Hymenochaetaceae* and *Gloeophyllaceae* families, were able to degrade the IMP is more surprising as their genomes contain very few or even no genes encoding cutinases (Pfam domain: PF01083; Supplementary Table 2). These observations augur the presence of new cutinases or polyurethanases yet to be uncovered. Furthermore, building on this work, we will tackle harder to degrade plastics in future studies.

Beyond providing functional leads and revealing fungal strains of interest, our study highlighted various hurdles underlying the maintenance and functional characterization of such a large collection. Notably, considerable research efforts are needed to address the difficulties of capture, isolation, and cultivation of fungal diversity from natural environments. Notably, we underscored the crucial role of strain authentication, in particular for freshly isolated strains as well as strains originating from ancient collections.

Our study proposes a methodological pipeline, exemplified by the heat-mapped numerical scoring system based on active vs non-active strain categorization (Fig. 5), which allows to evaluate the functional diversity at different taxonomic ranks. Nevertheless, we draw the attention toward the fact that the observed functional diversity is dependent on the inherent biological diversity of the tested taxonomic group. The seemingly broader functional diversity of Polyporales observed here (Fig. 5) should thus be interpreted with caution given the prevalence of this order in our fungal collection. Also, determining the appropriate number of strains to be screened to get a representative overview of the functional diversity at a given taxonomic rank is far from being obvious. For instance, our results demonstrated here that, to capture the largest functional diversity, one may need to assess up to 20 strains within a single species. In the same vein, the “strain effect” should not be overlooked, as illustrated by the numerous outliers frequently observed within each fungal taxonomic group, which may represent promising strains.

In a nutshell, searching for novel fungal biocatalysts adapted to new biotech applications requires an a priori-free screening covering a large diversity, both at different taxonomic ranks and at the intraspecies level. We foresee that fungal BRCs, by sampling in accordance to the Nagoya protocol (access to resources and benefit-sharing)^52,53^, and preserving large taxonomic and intraspecies diversity, along with evolving screening methods, can play a major role in the discovery and engineering of bio-inspired solutions to address the various challenges of this century.

## Methods

### Chemicals and reagents

Reactive Black (RB5; dye content 50%), lignosulfonic acid sodium salt (LGS; purity 90%), and Avicel PH-101 (AVI) were purchased from Sigma-Aldrich (Saint-Quentin-Fallavier, France). Basic Blue 41 (BB41, dye content 40%) was purchased from Setaş company (Turkey) and Impranil DLN-SD (IMP, purity 40%) from Covestro (Leverkusen, Germany). Malt extract was purchased from Duchefa Biochemie (Haarlem, Netherlands), agar from Becton Dickinson (Grenoble, France), and YNB from Fischer Scientific (Illkirch, France). Most other chemicals were purchased from Sigma-Aldrich unless stated otherwise.

### Collection of fungi and deposit of strains in the CIRM-CF

The CIRM-CF collection was established in 2006, resulting from the fusion of the collection of filamentous fungi of agro-industrial interest already held by our laboratory (BBF) and existing collections from different organizations: the French National Research Institute for Agriculture, Food and Environment (INRAE), the National Museum of Natural History (MNHN), the French Agricultural research and international cooperation organization (CIRAD), and the French Universities of Pharmacy (Paris Descartes, UGA Université Grenoble-Alpes). Since 2007, the CIRM-CF collection is regularly enriched and diversified via field-collecting in natural habitats, carried out in mainland France, and also in overseas territories, i.e., Guadeloupe, Martinique, French New Caledonia, and French Guiana. The initial identification of field-collected fungi, based on macro- and micro-morphological features, is carried out by our consortium of expert mycologists from Universities and learned societies.

### Strain isolation, authentication, and preservation

Following initial identification (vide supra), isolation were performed by transferring tissues from collected sporocarps onto agar plates containing either malt agar 2% (MA2; malt extract, Duchefa Biochemie, Haarlem, Netherlands) for basidiomycetes or potato dextrose agar (PDA; Sigma-Aldrich, Saint-Quentin-Fallavier, France) for ascomycetes, and both supplemented with antibiotics (0.025% (w/v) Chloramphenicol and 0.04% Gentamicin). Then, at least three successive subcultures were performed using the same media without antibiotics to obtain visibly pure cultures.

To check the identity of candidate strains, genomic DNA was extracted from approximately 100 mg of fresh mycelia using the NucleoSpin PlantII Kit (Macherey–Nagel, France). Then, for each strain, at least one barcoding gene (mainly rDNA-ITS) or species-specific genes were amplified by PCR. To this end, the following primers were used: ITS1/ITS4 and ITS5/ITS4 for rDNA-ITS amplification^54^, TEF1α-983-F-CF2/TEF1α-2218-R-CR2 for TEF-1α region amplification^55^. Following a protocol adapted from Lomascolo et al.^56^, the PCR reaction (50 µL total volume) contained 50 ng genomic DNA and 1.5 U Expand™ High Fidelity PCR master mix and primers (0.3 µM) (Roche, France). DNA amplification was then performed in a Mastercycler Nexus GSX1 (Eppendorf, Montesson, France) using the following sequence: 1 cycle at 94 °C for 2 min; 10 cycles of 94 °C for 30 s/55 °C for 90 s/72 °C for 1 min; 20 cycles of 94 °C for 90 s/*T*_annealing_°C for 90 s/72 °C for 1 min; then 1 cycle at 72 °C for 7 min. Annealing temperatures (*T*_annealing_) were 51 °C (for ITS1/ITS4 amplification), 53 °C (for ITS5/ITS4 amplification), and 51 °C (for TEF1α-983-F-CF2/TEF1α-2218-R-CR2 amplification). The PCR products were sequenced by Genewiz (Leipzig, Germany). For the identification of *A. bisporus* and *Fusarium* strains, we used species-specific primers (all primers are provided in Supplementary Table 3) to probe the presence of species-specific genes by qPCR (CFX96 Touch Real-Time PCR, Bio-Rad, Marnes-la-Coquette, France). The qPCR reaction (20 µL total volume) contained 50 ng genomic DNA and 10 µL SsoAdvanced™ Universal SYBR® Green Supermix (Biorad, Marnes-la-coquette, France). The thermocycle was conducted according to the manufacturer’s recommendation.

Strains were entered in the CIRM-CF collection only upon agreement of both the molecular authentication and identification based on macro- and micro-morphological features. Subsequently, the authenticated strains were maintained using 3 storage modes, including cryo-conservation (under vapor phase of liquid nitrogen at −196 °C), water, and oil-submerged agar slant (at 4 °C). The most appropriate storage mode being species-dependent, the viability of stored strains was systematically controlled after 6 months post-entry in the collection. This know-how is available to the scientific community, through the CIRM-CF website (https://www.cirm-fungi.fr).

### Multiphenotyping assay

The 1031 assessed strains were selected in the CIRM-CF collection with the objective to not only cover the taxonomic diversity but also fairly represent the proportion of strains available within the different fungal families. For precultures, all fungal strains were cultivated in petri dishes containing either PDA or MA2 for ascomycetes or basidiomycetes, respectively. The preculture plates were incubated between 7 and 15 days, at 25 °C or 30 °C (see CIRM-CF website for details). For each strain, the preculture was used to inoculate a 6-well plate (Greiner bio-one, Dutscher, Brumath, France), in which each well contained 6 mL of minimum medium (1.5 % (w/v) of agar supplemented by YNB (6.7 g/L final)) and one of the following mixtures: 1.5% (w/v) of malt extract (well #1, positive growth control); 0.5% (w/v) of malt extract supplemented with 0.02% (w/v final concentration) of either RB5 (well #2) or BB41 (well #3); 0.5% (v/v) IMP (well #4); 1.5% (w/v) LGS (well #5); 1.5% (w/v) AVI (well #6). In parallel, for each strain we prepared a “negative” control culture on minimum medium agar-YNB. RB5 and BB41 solutions were filter-sterilized before addition to autoclaved agar-malt medium. Note that we added malt in RB5 and BB41 wells as otherwise no fungal growth could be observed. All media had a similar pH (ca. pH 5). Inoculation of each well was done by placing a mycelia-overgrown agar plug (of standardized size, 0.3 × 0.3 cm) into the center of a well. After inoculation, the plates were incubated during 13 days at 25 °C. As shown in Fig. 2, a scoring rule (from 0 to 4) was defined to estimate growth and functional phenotypes after 13 days of culture. Growth was estimated by visual comparison of growth diameter and density of mycelia with culture on agar-YNB plate (“negative” control, score = 0) and with culture on malt agar plate (positive control, score = 4). Examples of extreme scores (0 and 4) are shown in Fig. 2a. Note that dyes biosorption was not considered as decolorization. As the scoring method can be subjective, all the measures were collected by only two inter-trained persons.

### Statistics and reproducibility

To investigate the significance of differences between fungal families on each target compound, non-parametric Kruskal–Wallis test and Wilcoxon signed-rank test were performed using the R package. First, for each fungal family/target compound couples, the statistical data (mean, median, standard deviation, quartiles) were computed. Kruskal–Wallis test was performed to obtain a *p* value indicating the significant differences between all families. As the samplings were different between fungal families, the size effects are always large to moderate. To circumvent this effect, family pairwise comparison was further carried out with Wilcoxon signed-rank tests. Statistical information is given in Supplementary Data 3.

### Reporting summary

Further information on research design is available in the Nature Research Reporting Summary linked to this article.

## Supplementary information

Supplementary information

Description of Supplementary Files

Supplementary Data 1

Supplementary Data 2

Supplementary Data 3

Reporting Summary

## Data Availability

All data are available within the article and its Supporting Information File and from the corresponding author upon reasonable request. A reporting summary for this Article is available as a Supplementary Information file. Supplementary Data files are available online on the FigShare platform (10.6084/m9.figshare.14742810). The CIRM-CF (“Centre International de Ressources Microbiennes – Champignons Filamenteux”) repository can be freely accessed at https://www.cirm-fungi.fr.
