# Supplementary material for: Large-scale phenotyping of 1,000 fungal strains for the degradation of non-natural, industrial compounds
Source: Commun Biol. 2021 Jul 15;4:871. doi: 10.1038/s42003-021-02401-w (PMC8282864; doi:10.1038/s42003-021-02401-w)
Supplement: Supplementary file 1 — Supplementary information [file 42003_2021_2401_MOESM1_ESM.pdf]

SUPPLEMENTARY INFORMATION FOR

Large-scale phenotyping of 1,000 fungal strains for the degradation of non-natural, industrial compounds

David Navarro[1,2*], Delphine Chaduli[1,2], Sabine Taussac[1,2], Laurence Lesage-Messeen[1,2], Sacha Grisel[1], Mireille Haon[1], Philippe Callac[3], Régis Courtecuisse[4], Cony Decock[5], Joëlle Dupont[6], Florence Forget[3], Jacques Fournier[7], Jacques Guinberteau[3], Christian Lechat[8], Pierre-Arthur Moreau[4], Laetitia Pinson[3], Bernard Rivoire[9], Lucile Sage[10], Stéphane Welti[4], Marie-Noëlle Rosso[1], Jean-Guy Berrin[1], Bastien Bissaro[1*] and Anne Favel[1,2].

[1]INRAE, Aix Marseille Univ., BBF, UMR1163, 13009 Marseille, France
[2]INRAE, Aix Marseille Univ., CIRM-CF, 13009 Marseille, France
[3]INRAE, MYCSA, UR1264, 33882 Villenave d'Ornon, France
[4]Faculté de Pharmacie Lille, Université de Lille, LGCgE, ER4, F-59006, Lille, France.
[5]Mycothèque de l'Université Catholique de Louvain (MUCL), Earth and Life Institute, Microbiology, B-1348 Louvain-la-Neuve, Belgium.
[6]Institut de Systématique, Evolution et Biodiversité, ISYEB – UMR 7205 – CNRS, MNHN, UPMC, EPHE, Muséum national d'histoire naturelle, Sorbonne Universités, 57 rue Cuvier, CP39, 75005 Paris, France
[7]Las Muros, Rimont, 09420, France
[8]Ascofrance, F-79360 Villiers-en-Bois, France.
[9]Société Linnéenne, 69006 Lyon, France
[10]Université Grenoble Alpes, LECA, UMR UGA-USMB-CNRS 5553, CS 40700 38058 Grenoble, France.

* corresponding authors: David Navarro (david.navarro@inrae.fr) and Bastien Bissaro (bastien.bissaro@inrae.fr)

This file includes:

1. Supplementary Figures
2. Supplementary Tables
3. Supplementary References

1. Supplementary Figures

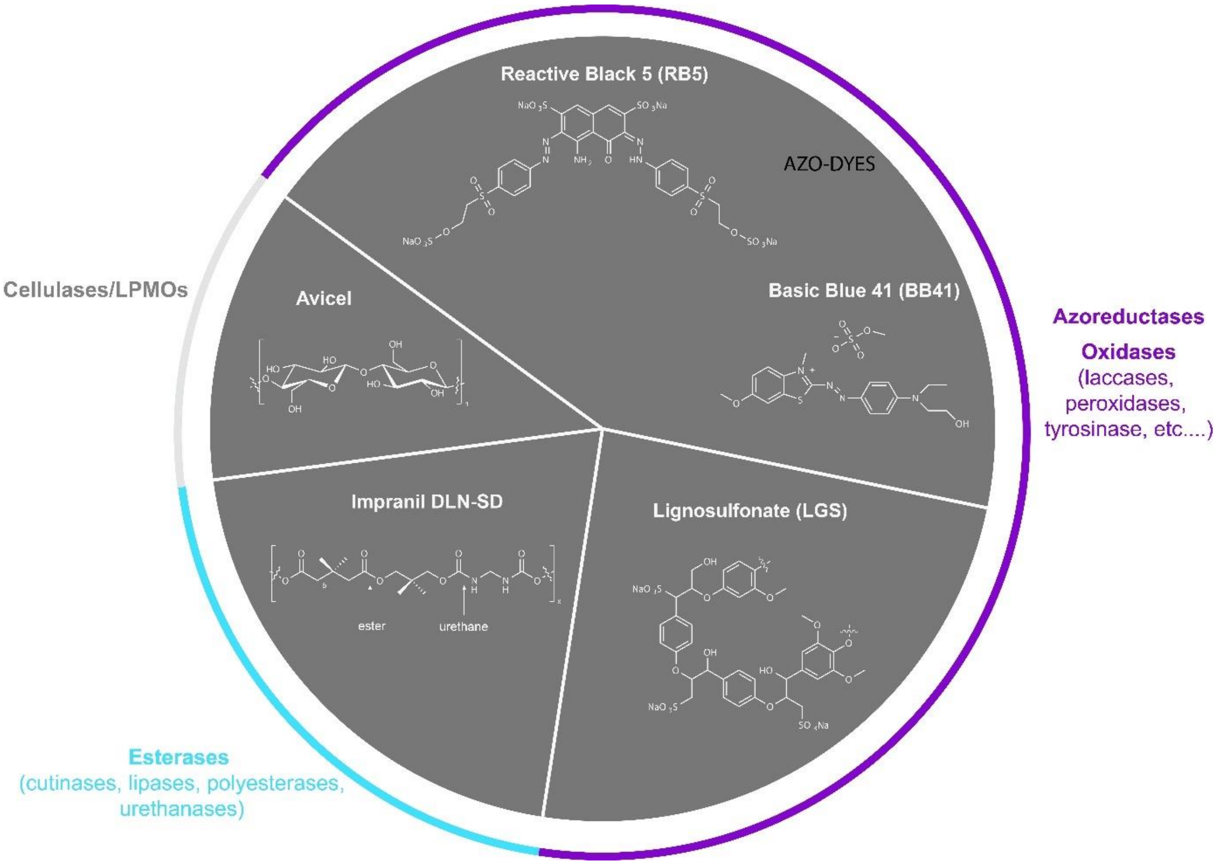

Supplementary Fig. 1. Chemical structures of the 5 targeted non-natural, industrial compounds. Enzymatic activities suggested to be involved in degradation of the different compounds are indicated in the figure.

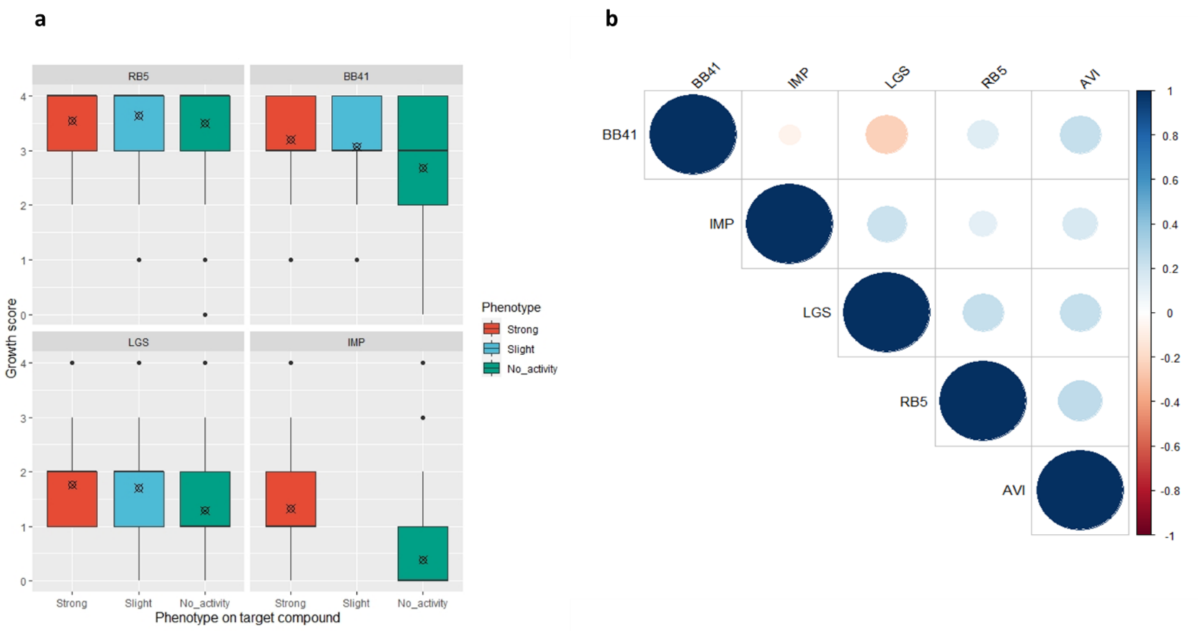

Supplementary Fig. 2. Correlation analysis between (a) growth and functional phenotypes and (b) between the functional phenotypes. In panel a, the box plots show the distribution of growth scores (y-axis) for each phenotype levels: strong (scores 4-3; red color), slight (scores 2-1 ; blue color) and inactive (score 0 ; green color). Boxes show the interquartile range (i.e. data comprised between the first and last quartile, 25<x<75%), black bars show the median and crossed-circles show the mean. The figure shows clearly that there is no correlation between growth and functional phenotypes. In panel b, the color scale on the right-hand side indicates the correlation coefficient, from 1 (perfect positive correlation) to -1 (perfect negative correlation). The diameter of circles represents the absolute value of correlation for each pair of functional phenotypes. The figure shows the absence of correlation in between most functional phenotypes. Raw data are available in the **Supplementary Data 2**.

2. Supplementary Tables

Supplementary Table 1. Overview of the taxonomic diversity of phenotyped strains.

Order	Family	Number of families	Number of genera	Number of species	Number of strains
Basidiomycota	**Agaricales**	**15**	**33**	**58**	**181**
	Boletales	5	5	6	6
	Atheliales	1	1	1	1
	**Polyporales**	**9**	**68**	**126**	**392**
	**Russulales**	**6**	**10**	**13**	**27**
	**Gloeophyllales**	**1**	**4**	**8**	**20**
	Corticiales	1	1	1	2
	**Hymenochaetales**	**2**	**8**	**15**	**49**
	Cantharellales	2	4	4	9
	Microstromatales	1	1	1	1
Ascomycota	**Eurotiales**	**2**	**4**	**22**	**66**
	Chaetothyriales	1	1	1	1
	**Pleosporales**	**4**	**9**	**9**	**18**
	Capnodiales	1	3	3	4
	Dothideales	1	1	1	1
	Helotiales	1	3	2	4
	Chaetomellales	1	1	1	1
	**Hypocreales**	**8**	**46**	**73**	**147**
	Coniochaetales	1	1	1	1
	Sordariales	3	3	3	3
	Pezizales	1	1	3	3
	**Xylariales**	**5**	**16**	**41**	**83**
	Rhinotrichum spp.				1
	Saccharomycetales	2	2	1	2
Mucoromycota	Mucorales	2	3	4	6
	Umbelopsidales	1	1	1	1
	Mortierellales	1	1	1	1
		78	231	400	1031

Supplementary Table 2. Occurrence of the PF01083 domain (cutinase domain) in the sequenced fungal genomes available in the JGI Mycocosm portal (Grigoriev *et al*., 2011; Grigoriev *et al*., 2014)[1,2]

Fungal families	Number of PF01083 domain "cutinase"	Number of sequenced genome
**Ascomycota**	6966	1070
Eurotiales	1448	279
Pleosporales	987	93
Hypocreales	897	98
Xylariales	793	113
**Basidiomycota**	1117	551
Agaricales	301	164
Gloeophyllales	0	4
Hymenochaetales	0	14
Polyporales	2	77
Russulales	12	40
**Mucoromycota**	0	97

Supplementary Table 3. List of primers used for the molecular authentication of candidate strains.

References	gene fragment	species	Forward		Reverse	
Stielow *et al*., 2015 [3]	TEF-1α	*Ascomycetes*	TEF1α-983-F	GCYCCYGGHCAYCGTGAYTTYAT	EF1-1567R	ACHGTRCCRATACCACCRATCTT
Rehner *et al*., 2005 [4]	TEF-1α	*Basidiomycetes*	TEF1α-983-F-CF2	GCYCCYGGHCAYCGTGAYTTYAT	TEF1α-2218-R-CR2	ATGACACCRACRGCRACRGTYTG
White *et al*., 1990 [5]	ITS1-5,8S-ITS2	*Ascomycetes*	ITS5	GGAAGTAAAAGTCGTAACAAGG	ITS4	TCCTCCGCTTATTGATATGC
	ITS1-5,8S-ITS2	*Basidiomycetes and Mucoromycetes*	ITS1	TCCGTAGGTGAACCTGCGG	ITS4	TCCTCCGCTTATTGATATGC
Parry and Nicholson, 1996 [6]	RAPD marker	*Fusarium poae*	Fp82F	CAAGCAAACAGGCTCTTCACC	Fp82R	TGTTCCACCTCAGTGACAGGTT
Nicholson *et al*., 1998 [7]	RAPD marker	*Fusarium culmorum*	Fc01F	ATGGTGAACTCGTCGTGGC	Fc01R	CCCTTCTTACGCCAATCTCG
	RAPD marker	*Fusarium graminearum*	Fg16NF	ACAGATGACAAGATTCAGGCACA	Fg16NR	TTCTTTGACATCTGTTCAACCCA
Yoder et Christianson, 1998 [8]	RAPD marker	*Fusarium crookwellense*	FcroF	CTCAGTGTCCACCGCGTTGCGTAG	FcroR	CTCAGTGTCCCATCAAATAGTCC
Wilson *et al*., 2004 [9]	RAPD marker	*Fusarium sporotrichioides*	FsporF1	CGCACAACGCAAACTCATC	LansporR1	TACAAGAAGACGTGGCGATAT
Turner *et al*., 1998 [10]	RAPD marker	*Fusarium avenaceum*	JIAf	GCTAATTCTTAACTTACTAGGGGCC	JIAr	CGTTAATAGGTTATTTACATGGGCG
Moller *et al*., 1999 [11]	RAPD marker	*Fusarium subglutinans*	61-2 F	GGCCACTCAAGAGGCGAAAG	61-2 R	GTCAGACCAGAGCAATGGGC
Picot *et al*., 2012 [12]	FUM 1	*Fusarium proliferatum*	FproF	TGCTCGTCATCCCTGATAG	FproR	GAAGATGGCATTGATTGCCTC
Aguado *et al*., 2018 [13]	FUM 1	*Fusarium verticillioides*	Fum1-654	CGGTTGTTCATCATCTCTGA	Fum1-1158	GCTCCCGATGTAGAGCTTGTT
Scauflaire *et al*., 2012 [14]	EF1- α	*Fusarium temperatum*	FtemF	AAGACCTGGCGGGC	FtemR	TCAGAAGGTTGTGGCAATGG
This study	ITS2	*Agaricus bisporus var. bisporus*	5,8 S 3'F	AYGCCTGTYYGAGYGTC	Abisp ITS2	TGTCCTTAGACGATTAGAAGC

3. Supplementary References

1. Grigoriev, I. V. *et al.* Fueling the future with fungal genomics. *Mycology* **2**, 192–209 (2011).
2. Grigoriev, I. V. *et al.* MycoCosm portal: gearing up for 1000 fungal genomes. *Nucleic Acids Res.* **42**, D699–D704 (2014).
3. Stielow, J. B. *et al.* One fungus, which genes? Development and assessment of universal primers for potential secondary fungal DNA barcodes. *Persoonia Mol. Phylogeny Evol. Fungi* **35**, 242–263 (2015).
4. Rehner, S. A. & Buckley, E. A Beauveria phylogeny inferred from nuclear ITS and EF1-alpha sequences: evidence for cryptic diversification and links to Cordyceps teleomorphs. *Mycologia* **97**, 84–98 (2005).
5. White, T. J., Bruns, T., Lee, S. & Taylor, J. AMPLIFICATION AND DIRECT SEQUENCING OF FUNGAL RIBOSOMAL RNA GENES FOR PHYLOGENETICS. in *PCR Protocols* 315–322 (Elsevier, 1990).
6. Parry, D. W. & Nicholson, P. Development of a PCR assay to detect Fusarium poae in wheat. *Plant Pathol.* **45**, 383–391 (1996).
7. Nicholson, P. *et al.* Detection and quantification of *Fusarium culmorum* and *Fusarium graminearum* in cereals using PCR assays. *Physiol. Mol. Plant Pathol.* **53**, 17–37 (1998).
8. Yoder, W. T. & Christianson, L. M. Species-specific primers resolve members of *Fusarium* section *Fusarium*. Taxonomic status of the edible 'Quorn' fungus reevaluated. *Fungal Genet. Biol. FG B* **23**, 68–80 (1998).
9. Wilson, A., Simpson, D., Chandler, E., Jennings, P. & Nicholson, P. Development of PCR assays for the detection and differentiation of *Fusarium sporotrichioides* and *Fusarium langsethiae*. *FEMS Microbiol. Lett.* **233**, 69–76 (2004).
10. Turner, A. S., Lees, A. K., Rezanoor, H. N. & Nicholson, P. Refinement of PCR-detection of *Fusarium avenaceum* and evidence from DNA marker studies for phenetic relatedness to *Fusarium tricinctum*. *Plant Pathol.* **47**, 278–288 (1998).
11. Möller, E. M., Chełkowski, J. & Geiger, H. H. Species-specific PCR Assays for the Fungal Pathogens *Fusarium moniliforme* and *Fusarium subglutinans* and their Application to Diagnose Maize Ear Rot Disease. *J. Phytopathol.* **147**, 497–508 (1999).
12. Picot, A. *et al.* Interactions between *Fusarium verticillioides* and *Fusarium graminearum* in maize ears and consequences for fungal development and mycotoxin accumulation. *Plant Pathol.* **61**, 140–151 (2012).
13. Aguado, A. *et al.* Priming to protect maize from *Fusarium verticillioides* and its fumonisin accumulation. *J. Sci. Food Agric.* **99**, 64–72 (2019).
14. Scauflaire, J., Godet, M., Gourgue, M., Liénard, C. & Munaut, F. A multiplex real-time PCR method using hybridization probes for the detection and the quantification of *Fusarium proliferatum*, *F. subglutinans*, *F. temperatum*, and *F. verticillioides*. *Fungal Biol.* **116**, 1073–1080 (2012).
