# Supplementary material for: Large-scale phenotyping of 1,000 fungal strains for the degradation of non-natural, industrial compounds
Source: Commun Biol. 2021 Jul 15;4:871. doi: 10.1038/s42003-021-02401-w (PMC8282864; doi:10.1038/s42003-021-02401-w)
Supplement: Supplementary file 2 — Description of Supplementary Files [file 42003_2021_2401_MOESM2_ESM.pdf]

Description of Additional Supplementary Files

File Name: Supplementary Data 1
Description: Origin and authentication of the 1,031 selected strain.

File Name: Supplementary Data 2
Description: Raw data of the growth and functional phenotyping.

File Name: Supplementary Data 3
Description: Statistical analysis of the distribution of phenotypic scores at the family level.
