# Supplementary material for: Large-scale phenotyping of 1,000 fungal strains for the degradation of non-natural, industrial compounds
Source: Commun Biol. 2021 Jul 15;4:871. doi: 10.1038/s42003-021-02401-w (PMC8282864; doi:10.1038/s42003-021-02401-w)
Supplement: Supplementary file 6 — Reporting Summary [file 42003_2021_2401_MOESM6_ESM.pdf]

nature research

Corresponding author(s): David NAVARRO and Bastien BISSARO

Last updated by author(s): Jun 7, 2021

Reporting Summary

Nature Research wishes to improve the reproducibility of the work that we publish. This form provides structure for consistency and transparency in reporting. For further information on Nature Research policies, see our Editorial Policies and the Editorial Policy Checklist.

Statistics

For all statistical analyses, confirm that the following items are present in the figure legend, table legend, main text, or Methods section.

n/a	Confirmed	
☐	☒	The exact sample size (n) for each experimental group/condition, given as a discrete number and unit of measurement
☐	☒	A statement on whether measurements were taken from distinct samples or whether the same sample was measured repeatedly
☐	☒	The statistical test(s) used AND whether they are one- or two-sided<br>*Only common tests should be described solely by name; describe more complex techniques in the Methods section.*
☒	☐	A description of all covariates tested
☒	☐	A description of any assumptions or corrections, such as tests of normality and adjustment for multiple comparisons
☐	☒	A full description of the statistical parameters including central tendency (e.g. means) or other basic estimates (e.g. regression coefficient) AND variation (e.g. standard deviation) or associated estimates of uncertainty (e.g. confidence intervals)
☐	☒	For null hypothesis testing, the test statistic (e.g. F, t, r) with confidence intervals, effect sizes, degrees of freedom and P value noted<br>*Give P values as exact values whenever suitable.*
☒	☐	For Bayesian analysis, information on the choice of priors and Markov chain Monte Carlo settings
☒	☐	For hierarchical and complex designs, identification of the appropriate level for tests and full reporting of outcomes
☒	☐	Estimates of effect sizes (e.g. Cohen's d, Pearson's r), indicating how they were calculated

Our web collection on statistics for biologists contains articles on many of the points above.

Software and code

Policy information about availability of computer code

Data collection	No specific software was used for data collection
Data analysis	Data were analyzed with Excel and visualized with R Studio (v1.3.959) and/or Illustrator 2017 (Adobe).

For manuscripts utilizing custom algorithms or software that are central to the research but not yet described in published literature, software must be made available to editors and reviewers. We strongly encourage code deposition in a community repository (e.g. GitHub). See the Nature Research guidelines for submitting code & software for further information.

Data

Policy information about availability of data

All manuscripts must include a data availability statement. This statement should provide the following information, where applicable:

- Accession codes, unique identifiers, or web links for publicly available datasets
- A list of figures that have associated raw data
- A description of any restrictions on data availability

Provide your data availability statement here.

Field-specific reporting

Life sciences study design

All studies must disclose on these points even when the disclosure is negative.

Sample size	The sample size was dictated by the diversity of the fungal collection and practical feasability of screening > 1,000 strains.
Data exclusions	No Data were excluded. All data are displayed on graphs and/or provided in Tables.
Replication	All independent experiments pertaining to fungal growth and degradation were carried out once. The unprecedented very large number of assessed fungal strains did not allow replication. Yet, statistical analysis of the large number of generated data (> 12,000 data) compensate for the lack of replication, which does not affect the drawn conclusions (mainly based on trends).
Randomization	Two self-intertrained persons assessed randomly the strains spanning the phylogenetic tree.
Blinding	The assessed strains were identified by numbers (e.g., strain CIRM 1, strain CIRM 792, etc...), and not by their actual name, avoiding thereby potential biases during scoring of growth and degradation phenotypes.

Reporting for specific materials, systems and methods

We require information from authors about some types of materials, experimental systems and methods used in many studies. Here, indicate whether each material, system or method listed is relevant to your study. If you are not sure if a list item applies to your research, read the appropriate section before selecting a response.

Materials & experimental systems

n/a	Involved in the study
☒	☐ Antibodies
☒	☐ Eukaryotic cell lines
☒	☐ Palaeontology and archaeology
☐	☒ Animals and other organisms
☒	☐ Human research participants
☒	☐ Clinical data
☒	☐ Dual use research of concern

Methods

n/a	Involved in the study
☒	☐ ChIP-seq
☒	☐ Flow cytometry
☒	☐ MRI-based neuroimaging

Animals and other organisms

Policy information about studies involving animals; ARRIVE guidelines recommended for reporting animal research

Laboratory animals	No animals were used in the present study. Only filamentous fungi strains (see below)
Wild animals	No animals were used in the present study.
Field-collected samples	All details regarding field-collected samples are provided in the main text results and experimental sections of the manuscrit.
Ethics oversight	All field-collected samples are collected in accordance to the OECD Best Practice Guidelines for Biological Resource Centres

Note that full information on the approval of the study protocol must also be provided in the manuscript.
